# Dimethyl Sulfoxide: Morphological, Histological, and Molecular View on Developing Chicken Liver

**DOI:** 10.3390/toxics9030055

**Published:** 2021-03-12

**Authors:** Lenka Luptakova, Simona Dvorcakova, Zuzana Demcisakova, Lassaad Belbahri, Katarina Holovska, Eva Petrovova

**Affiliations:** 1 Department of Biology and Physiology, University of Veterinary Medicine and Pharmacy in Kosice, Komenskeho 73, 041 81 Kosice, Slovakia; dvorcakovasimona@gmail.com; 2 Department of Morphological Disciplines, University of Veterinary Medicine and Pharmacy in Kosice, Komenskeho 73, 041 81 Kosice, Slovakia; zuzana.demcisakova@gmail.com (Z.D.); katarina.holovska@uvlf.sk (K.H.); eva.petrovova@uvlf.sk (E.P.); 3 Laboratory of Soil Biodiversity, Department of Biology, University of Neuchatel, 2000 Neuchatel, Switzerland; lassaad.belbahri@unine.ch

**Keywords:** chicken embryo, cytochrome P450, dimethyl sulfoxide, development, liver, toxicity

## Abstract

Dimethyl sulfoxide (DMSO) is widely used as a solvent for small hydrophobic drug molecules. However, the safe volume allowing to avoid its embryotoxic effect has been poorly studied. In this study, we documented the effects of dimethyl sulfoxide (DMSO) in the developing chicken embryo at morphological, histological, and molecular levels. We focused on the developing chicken liver as the main organ involved in the process of detoxification. In our study, 100% DMSO was administered subgerminally onto the eggshell membrane (membrana papyracea) at various volumes (5, 10, 15, 20, 25, 30, 35, and 50 µL) on 4th embryonic day (ED). We focused on histopathological alterations of the liver structure, and noticed the overall impact of DMSO on developing chicken embryos (embryotoxicity, malformation). At the molecular level, we studied cytochrome P450 complex (*CYP*) isoform’s activities in relation to changes of *CYP1A5*, *CYP3A37*, and *CYP3A80* gene expression. Total embryotoxicity after application of different doses of DMSO on ED 4, and the embryo lethality increased with increasing DMSO amounts. Overall mortality after DMSO administration ranged below 33%. Mortality was increased with higher amounts of DMSO, mainly from 20 µL. The highest mortality was observed for the highest dose of DMSO over 35 µL. The results also showed a decrease in body weight with increased application volumes of DMSO. At the histological level, we observed mainly the presence of lipid droplets and dilated bile canaliculi and sinusoids in samples over the administration of 25 µL of DMSO. While these findings were not statistically significant, DMSO treatment caused a significant different up-regulation of mRNA expression in all studied genes. For *CYP1A5*, *CYP3A37*, and *CYP3A80* DMSO volumes needed were 15 µL, 10 µL, and 20 µL, respectively. A significant down-regulation of all studied *CYP* isoform was detected after application of a DMSO dose of 5 µL. Regarding the morphological results, we can assume that the highest safe dose of DMSO without affecting chicken embryo development and its liver is up to 10 µL. This conclusion is corroborated with the presence of number of malformations and body weight reduction, which correlates with histological findings. Moreover, the gene expression results showed that even the lowest administered DMSO volume could affect hepatocytes at the molecular level causing down-regulation of cytochrome P450 complex (*CYP1A5*, *CYP3A37*, *CYP3A80*).

## 1. Introduction

Dimethyl sulfoxide (DMSO) is an organic polar aprotic molecule generally used in low concentration because of its medically useful properties. Its main properties are induction of anti-inflammation, nerve blockage, muscle relaxation, penetrating vehicle for various drugs as well as its action as a solvent for the dissolution of small hydrophobic drug molecules due to its amphipathic nature. Moreover, DMSO is also used in cell biology as inducer of cell differentiation, free radical scavenger and for cryopreservation [1,2]. DMSO is though commonly used in research, but unfortunately, there are no reported information about the used concentrations in numerous studies. In general, DMSO is accepted as a nontoxic agent below 10% (*v*/*v*) without no scientific support. In practice, it is supposed that DMSO effects are inconsiderable. The manifested effects of DMSO application are decisive for the dose and the route of application. Verheijen et al. 2019 published that LD50 value in monkey was 880 g when applied on the skin or 320 g when injected intravenously in 80 kg humans [2]. On the other side, it is well documented that DMSO is known to be cytotoxic at higher concentrations. Therefore, it is necessary to determine DMSO threshold concentrations for cells and organisms and to deepen our knowledge of DMSO effects at the molecular level. Unfortunately, very little information has been reported on the behavioural and developmental effects of DMSO. Chen et al. (2011) monitored the effects of 0.01, 0.1 and 1% DMSO on zebrafish embryo. No developmental defects were observed using 0.01 and 0.1% concentrations, but 1% DMSO induced severe deformity rates [3]. The excretion of DMSO is performed mostly through the kidneys, and small part is excreted by lungs and liver. Part of DMSO is metabolized to the volatile metabolite dimethyl sulfide having a characteristic garlic- or oyster-like smell after excretion. The occurrence of adverse reactions seems to be related to higher doses of DMSO, and it seems safe to continue the use of small doses of DMSO [4].

The P450 cytochrome families 1–3 are the major source of variability in drug pharmacokinetics and response. They have been described as the major xenobiotic-metabolizing enzymes and are involved in bioactivation or inactivation of numerous xenobiotic compounds, such as drugs and environmental chemicals [5,6]. Approximately 10,000 avian species are currently known, and are exposed to drugs, pesticides and other environmental chemicals. Therefore, their xenobiotic-metabolizing ability is an important field of research [5]. Similar to mammals, chickens have two *CYP1A* genes (*CYP1A4* and *CYP1A5*) which are orthologous to mammalian *CYP1A1* and *CYP1A2* respectively. The importance of chicken *CYP1As* in metabolism endogenous compounds and xenobiotics has been documented in many previous studies [7,8,9,10,11]. Cytochrome P450s *CYP1A1* and *CYP1A2* can metabolize a broad range of foreign compounds and drugs. Substrates for *CYP1A2*, mammalian orthologues of avian *CYP1A5* include drugs, industrial chemicals, and environmental toxicants [12]. The enzyme activity is variable due to a combination of genetic polymorphism and environmental factors affecting enzyme expression level and activity. The expression of *CYP1A2* is highly regulated by the aryl hydrocarbon receptor (AHR) [13]. *CYP3A* represent a family of P450 cytochromes involved in the metabolism of both endogenous and exogenous natural and synthetic compounds. Chicken *CYP1A5* has not only the catalytic activity, regulation, and detoxification role, but also the mechanism of T-2 biotransformation in chickens [14]. In the phase I metabolic enzymes of CYP3A, only *CYP3A37* and *CYP3A80* are present in birds in comparison to humans, where four different types of *CYP3A* can be found. In, chicken, *CYP3A37* is involved in the hydroxylation of steroids, such as progesterone and in N-demethylation of erythromycin. The chemical ketoconazole is known as an inhibitor of the metabolic activity of chicken *CYP3A37* as well as human *CYP3A4* [15,16,17]. Avian *CYP3A37* possesses similar catalytic and inhibitory properties than human *CYP3A4*. The tissue distribution of avian *CYP3A37* is similar to human *CYP3A4* with the highest level in the liver followed by the small intestine [18,19]. *CYP3A37* appears to be one of the most important genes for metabolism of xenobiotics [20]. Avian *CYP3A80* showed high similarity with human *CYP3A4* (59%) sequence identity [21]. Therefore, it is suggested that its expression can be induced by xenobiotics leading to the acceleration of their metabolism (autoinduction) or of concomitantly administered *CYP3A4* substrates/drugs, thereby significantly altering their pharmacokinetic and pharmacodynamic profiles [22]. The aim of three R’s principle (stands for Replacement, Reduction and Refinement) is to reduce the number of animals used, refine experiments so that the pain and suffering endured by an animal is kept to a minimum; to replace higher animals with lower organisms, and to replace potentially dangerous and harmful tests with alternative methods [23]. Chick embryos have been adopted as an alternative model for human biology and scientific research for many years dating back to around the 16th Century. Under national laws worldwide, the chicken embryo is not regarded as a living animal until day 17 of development [24] and hence is considered a more ethical choice for experimental purposes. The use of chicken embryo in research and experiments as a model is primarily due to chick embryo development being fairly similar to that of a mammal. It also has added benefits. For instance, chick embryos are cheap, readily available, and have a short incubation time allowing extensive research to be conducted rapidly [25,26]. Biological research has for a long time used oviparous species for investigation into the very early embryonic stages due to their availability and accessibility. Although the chick embryo is preferred today as a fundamental model, its capacity to provide a sterile environment and a high yield of the biopharmaceutical within the egg white have enabled additional arguments for its effective use [27]. Furthermore, chick embryos are ideal models for the evaluation of chemicals and biomaterials with the capacity to determine the teratogenic or embryotoxic characteristics of various agents [28,29,30].

Based on wide use of DMSO as a solvent mainly for various substances and drugs, which are tested on avian embryos, we evaluated biological effect of DMSO on chicken embryo and especially its developing liver as an important organ for detoxification. The aim of our study was to answer the question regarding the safe volume of DMSO for dissolving substances or drugs that can be applied to a chicken egg. To answer our question, we evaluated embryotoxicity, changes in histological structure of liver, and the molecular level of gene expression of selected isoforms of cytochrome P450 complex.

## 2. Materials and Methods

Fertilized Lohmann Brown chicken eggs were purchased from the hatching farm (LP Nitra A.S., Parovske Haje, Slovakia). The eggs were incubated with blunt end up in a forced-draft constant-humidity incubator at 37.5 °C with continuous rocking until embryonic day (ED) 4. Embryos that were growth retarded or dysmorphic at the time of treatment were excluded from further study. At incubation day 4, the eggs were windowed on the blunt end, and varied volumes of DMSO were applied directly over the embryos on the top of inner shell membrane (membrana papyracea). Control group received 50 µL of water for injection. The ranges of DMSO volumes as well as the total numbers of embryos are listed in Table 1. The windows were closed using insulation tape as described earlier [31], and the eggs were returned to a still draft incubator with the same temperature and humidity settings for re-incubation until the time of sampling (ED 9). At the time of sampling, the embryos were removed from the eggs using a crook, weighted, and examined under a dissecting microscope SZ 61 with digital Promicra camera for external (eye, beak, palate, body wall, limbs) and internal anomalies (gastrointestinal system, microdissection of the heart).

There is no need to request animal protocol approval for the chicken embryo as an experimental model in ovo as they are exempt from the horizontal legislation on the protection of animals used for scientific purposes (2010/63/EU), as well as applicable laws in the United States.

### 2.1. Histology

#### 2.1.1. Light Microscopy

Liver samples were used for histological examination and were processed by a standard histological technique. For fixation, 4% neutral formaldehyde was used, and samples were embedded in paraffin. Then 5–7 μm thick slides were stained with haematoxylin and eosin (HE) and examined under a light microscope Olympus CX 43 (Japan) and documented with a Promicra camera (Tokyo, Japan).

#### 2.1.2. Transmission Electron Microscopy

For transmission electron microscopy examination, tissue samples up to 1 mm^3^ were fixed in 3% glutaraldehyde (Sigma-Aldrich, Bratislava, Slovak Republic) and postfixed in 1% osmium tetroxide (both in 0.1 M cacodylate buffer, pH 7.3, Fluka Chemie AG, Buchs, Switzerland). After dehydration in acetone, they were transferred to propylene oxide and embedded in Durcupan^TM^ ACM (Sigma-Aldrich Chemie GmbH, Darmstadt, Germany). Sections of the specimen were cut using the ultramicrotome LKB Nova (Bromma, Sweden). Semi-thin sections (1 μm) were stained with toluidine blue and examined under the light microscope Olympus CX 43 (Tokyo, Japan) and documented with a Promicra camera (Japan). Ultrathin sections (60–90 nm) were double contrasted with 1% uranyl acetate and 0.3% lead citrate and examined under a Tesla BS 500 electron microscope (Tesla Brno, Brno, Czech Republic).

### 2.2. Molecular Analysis—RNA Extraction and RT qPCR

The liver tissue for molecular analysis was collected on the 9th ED. Total RNA was extracted from liver tissue using QIAshredder and total Rneasy Mini Kit from Qiagen (Qiagen, Germany) following manufacturer’s instructions including genomic DNA digestion using the RNase-free DNase set (Qiagen, Germantown, MD, USA). The RNA purity and yields were analyzed using the NanoDrop Lite Spectrophotometer (Thermo Fisher Scientific, Waltham, MA, USA). We used two-step RT-qPCR approach. In the first step, complementary DNA (cDNA) synthesis was performed using protocol for High-Capacity cDNA Reverse Transcription Kit (Applied Biosystem, Waltham, MA, USA). 1 µg of total RNA was used to prepare 20 µL of cDNA that was then used for qPCR. In the second step, the quantification of genes of interest in the cDNA samples was performed using specific primers for *CYP1A5*, *CYP3A37* [33] and Taqman probe for *CYP3A80*. For *CYP1A5* and *CYP 3A37* we used SYBR Green Mastermix (Qiagen, USA) in a total volume of 25 µL. For *CYP3A4* we used TaqMan™ Gene Expression Assay (FAM; Applied Biosystem, USA). PCR mixture contained specific primers for each gene (*CYP1A5*, *CYP3A37*, *CYP3A80*; 300 nM), SYBR Green PCR MasterMix and water. cDNA for ubiquitin was used as endogenous control for calculating fold differences in RNA levels of cells treated vs not treated by DMSO using the 2^−ΔΔCT^ method. We included calculation of average Ct value for gene triplicates of each sample followed by the calculation of ΔCt and ΔΔCt for each sample. The last step included calculation of fold gene expression value 2^−ΔΔCT^. Each sample was analyzed as triplicates. qPCR was performed under same conditions for SYBR Green and Taqman with following steps: Initialization at 95 °C for 10 min., amplification for 40 cycles at 95 °C for 15 s followed by 60 °C for 1 min. Dissociation curve analysis was performed after each completed PCR run to ascertain the absence of nonspecific amplifications. The gene expression data were calculated against ubiquitin housekeeping gene and expression level of selected genes were normalized to untreated samples (control). Gene expression values were log transformed, because untransformed gene expression values will most likely not be normally distributed.

### 2.3. Statistical Analysis

Statistical analyses of data were performed using one-way ANOVA followed by multiple Dunnett’s test (GraphPad Prism 6.0). Results are expressed as a mean ± SD (standard deviation), and values of *p* < 0.01 were considered statistically significant.

## 3. Results

### 3.1. Total Embryotoxicity

**Figure 1:**
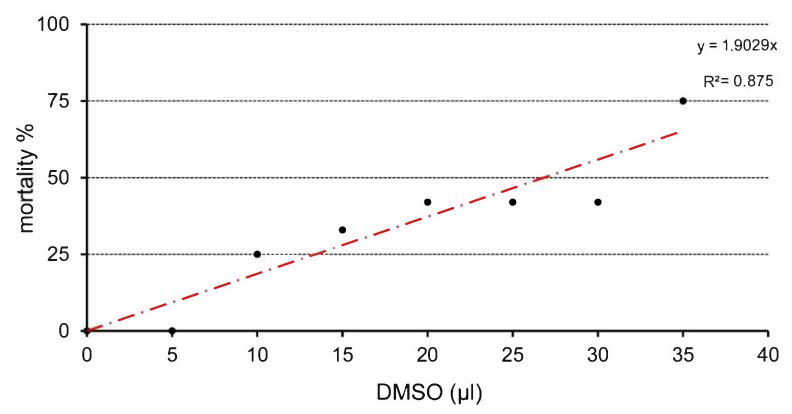
Relation between application dose of DMSO and mortality of chick embryos.

**Figure 2:**
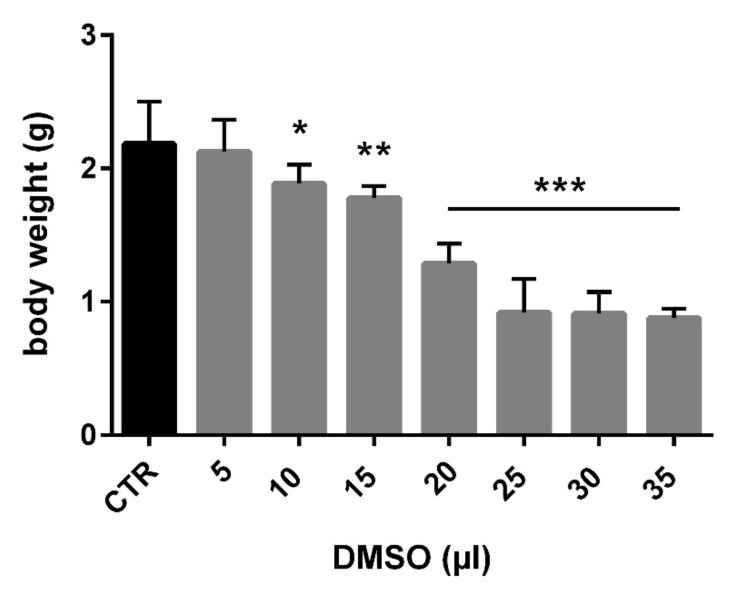
Relation between application dose of DMSO and body weight of chick embryo (CTR—control, * *p* < 0.01; ** *p* < 0.001; *** *p* < 0.0001).

Total embryotoxicity of different doses of DMSO injected on ED 4, was investigated at ED 9. The embryo lethality increased with the dose of DMSO (Figure 1). The mortality average after DMSO administration ranged under 33%. Mortality was increased, mainly from 20 µL. The highest mortality was observed in relation to the highest dose of DMSO (35 µL). It represents 75% dead embryos from this group (Table 1). Table 1 lists the wet weight of sampled embryos on ED 9 after DMSO different volume administration on ED 4 compared with the control group. In general, administration of DMSO resulted in decrease of embryonic body weight, with a clear correlation with the dose. Body weight was significantly decreased from administration of 10 µL DMSO (Figure 2). The malformations were observed during administration of higher amount of DMSO (25–35 µL) compared with the control group, with overall frequency below 16% (Table 1). Examples of malformations included opening body wall, anophthalmia, malformations of the limb bud, and general growth retardation. Haemorrhages were observed as well from 15 µL DMSO application. No specific pattern of malformations was observed among the treated embryos, irrespective of the dose.

**Figure 3:**
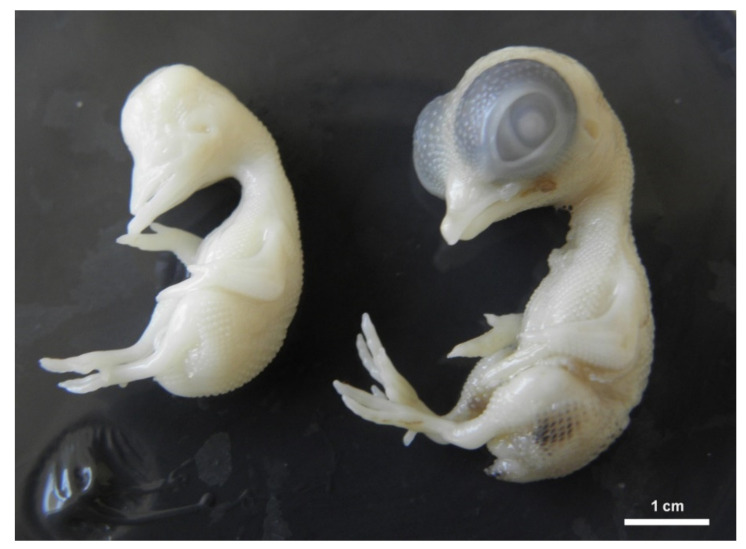
Chicken embryo development on ED 9. Right position: physiological development, left position: general growth retardation with anophthalmia.

**Figure 4:**
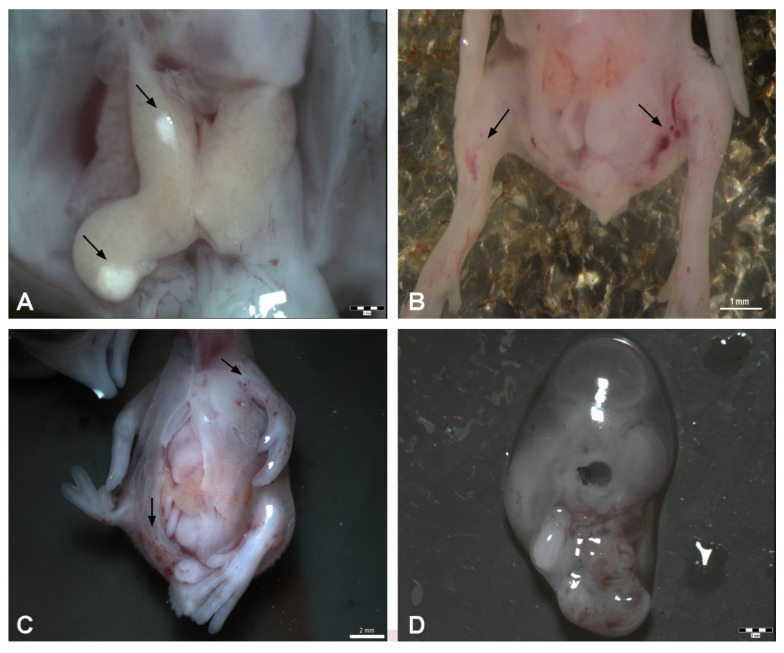
Malformations appearing after DMSO administration on ED 9. (**A**): Observation of local macroscopic colour changes on the liver after 10 μL of DMSO administration, scale bar 1 mm, (**B**): Haemorrhages on the limb buds after 15 μL of DMSO administration—arrows, scale bar: 1 mm, (**C**): Opening of the body wall, haemorrhages on the left-wing bud and pelvic region (arrows), malformation of the right limb bud—20 μL of DMSO administration, scale bar: 2 mm, (**D**): Growth retardation–25 μL of DMSO administration, scale bar: 2 mm.

Macroscopic observation revealed no changes in the size or shape of the liver. The organs were yellow, with shiny surface and the sections showed preservation of characteristic liver structure. However, we observed local macroscopic colour changes on the liver after 10 µL DMSO application in only one embryo (Figure 3 and Figure 4).

### 3.2. Histology

#### 3.2.1. Light Microscopy

**Figure 5:**
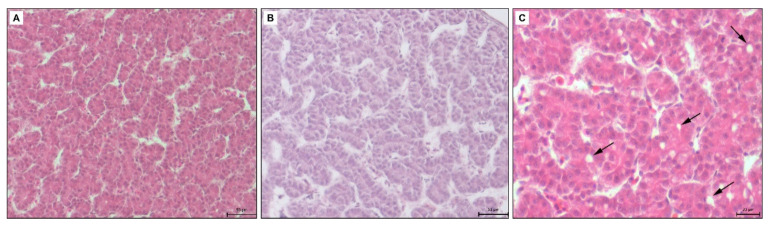
(**A**): Microphotograph of the chicken liver in control group, H-E staining, scale bar: 50 μm, (**B**): Microphotograph of the chicken liver in experimental group—5 μL of DMSO administration, H-E staining, scale bar: 50 μm, (**C**): Microphotograph of the chicken liver in experimental group—10 μL of DMSO administration, black arrows—dilated bile canaliculi, H-E staining, scale bar: 20 μm.

In the control group (HE), the liver had a classic morphological appearance. Hepatocytes of cylindrical or pyramidal shape had a round euchromatic nuclei with evident nucleoli. Their cytoplasm was strongly acidophilic. The cells were arranged in anastomosing two-cell thick cords or clusters (tubules). Their enlarged part (the vascular pole) was oriented towards the liver sinusoids and their narrowed part (the biliary pole) formed the bile canaliculus. The hepatic sinusoids had an irregular lumen and were lined with endothelial cells (Figure 5A).

**Figure 6:**
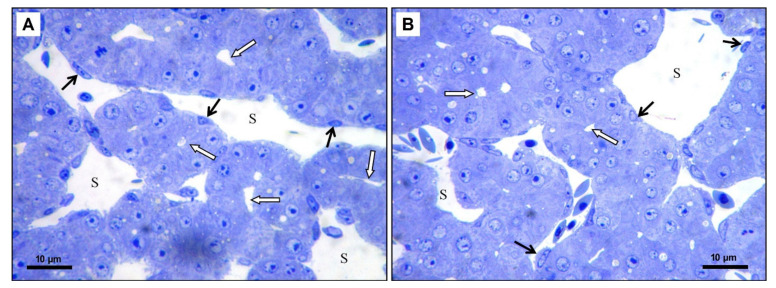
(**A**): Microphotograph of the chicken liver in experimental group—25 μL of DMSO administration, scale bar: 10 μm, (**B**): Microphotograph of the chicken liver in experimental group—50 μL of DMSO administration, scale bar: 10 μm; S—liver sinusoid, black arrows—endothelial cells, white arrows—dilated bile canaliculi, Toluidine blue staining.

In the experimental group 5 μL (5 DMSO-HE) the structure of the liver was unchanged. The size and shape of hepatocytes was preserved but the liver sinusoids were slightly dilated (Figure 5B). In the experimental group 10 μL (10 DMSO-HE) the structure of the liver was comparable to the previous group. In some parts of liver, the bile canaliculi were moderately dilated (Figure 5C). In the experimental group 25 μL (25 DMSO—toluidine blue), some hepatocytes contained a few small lipid droplets. The bile canaliculi were dilated with irregular lumen (Figure 6A). In the experimental group 50 μL (50 DMSO—toluidine blue), the structure of the liver was not changed markedly. Hepatocytes contained small lipid droplets, and the bile canaliculi and liver sinusoids were dilated (Figure 6B).

#### 3.2.2. Transmission Electron Microscopy

**Figure 7:**
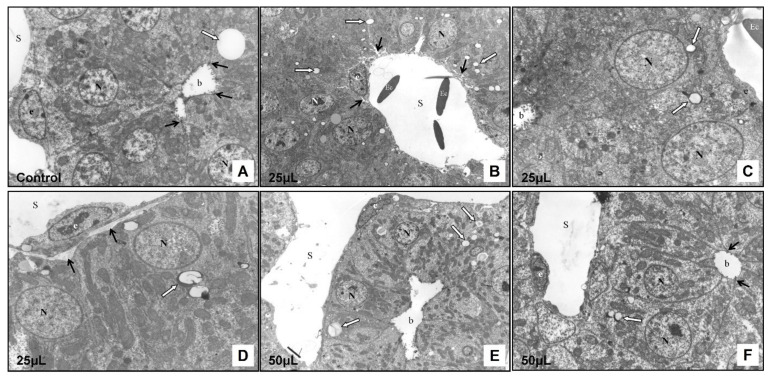
Transmission electron microscopy of the chicken developing liver. (**A**): Control group, magnification: 6500×; (**B**): Experimental group—25 μL of DMSO administration, magnification: 4000×; (**C**): Experimental group—25 μL of DMSO administration, magnification: 7500×; (**D**): Experimental group—25 μL of DMSO administration, magnification: 6800×; (**E**): Experimental group—50 μL of DMSO administration, magnification: 2800×; (**F**): Experimental group—50 μL of DMSO administration, magnification: 5100×; N—nucleus of hepatocyte, S—sinusoid, e—endothelial cell, Ec–erythrocyte, b—bile canaliculus, white arrow—lipid droplet, black arrows—intercellular junctions (**A**,**F**), black arrows—space of Disse (**B**,**D**).

The control group showed normal liver ultrastructure. In the cytoplasm of hepatocytes, there were numerous, well-developed mitochondria of oval or rod-shaped forms. The rough endoplasmic reticulum was located close to the mitochondria. Occasionally, lipid droplets were observed. The cytoplasm of the hepatocytes at the bile pole formed microvilli that protruded into the lumen of the bile canaliculus. The intercellular junctions between adjacent hepatocytes were clearly visible. At the vascular pole of hepatocytes, the cytoplasmic membrane was smooth without microvilli. The endothelial cells were in direct contact with hepatocytes and the Disse’s spaces were not formed between these cells (Figure 7A).

**Figure 8:**
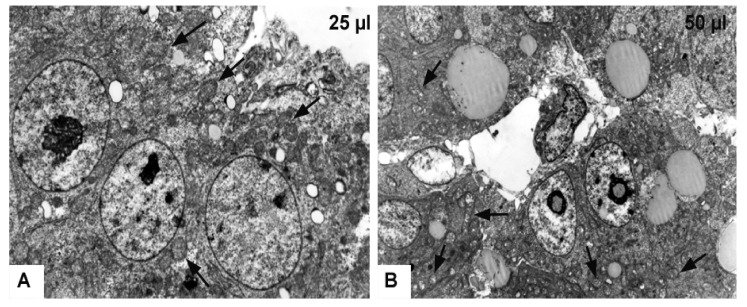
Transmission electron microscopy of the chicken developing liver. (**A**): Experimental group—25 μL of DMSO administration, magnification: 8800×; (**B**): Experimental group—50 μL of DMSO administration, magnification: 4400×; arrows—slightly dilated mitochondria (**A**), fragmented cristae of mitochondria with electron-lucent matrix (**B**).

In the experimental group 25 μL (25 μL DMSO) the ultrastructure of the cells was preserved. In the cytoplasm of the hepatocytes there were small lipid droplets. A narrow electron-dense line surrounded some of them. The bile canaliculi were moderately dilated but the intercellular junctions between adjacent cells and ultrastructure of the macrovilli remained intact. In some hepatocytes, mitochondria were slightly dilated, while in other cells these organelles were not affected (Figure 8A). In some areas, the space of Disse was formed between hepatocytes and endothelial cells. No changes were observed in the ultrastructure of the endothelial cells (Figure 7B–D).

In the experimental group 50 μL (50 μL DMSO) the hepatocytes contained lipid droplets of various shapes and sizes, which were surrounded by electron-dense line. Mitochondria were severely swollen with an electron-lucent matrix, and with fragmented cristae (Figure 8B). The bile canaliculi were dilated with short and irregular microvilli. However, the space of Disse between hepatocytes and endothelial cells was not formed (Figure 7E,F). Histological results showed alterations after the DMSO application in various volumes without statistical signification.

### 3.3. Gene Expression

**Figure 9:**
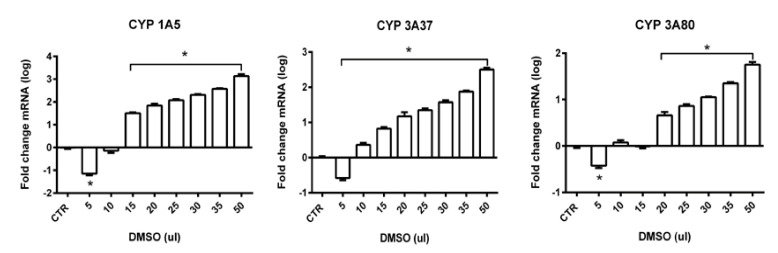
Relative gene expression from RT-qPCR analysis for genes *CYP1A5*, *CYP3A37* and *CYP3A80* (* statistically significant difference, *p* < 0.01; log-log transformed).

As endogenous control, the ubiquitin gene was used based on the analysis of gene expression stability using GeNorm algorithm. Ubiquitin was the most stable in all tested samples. A low M-value 0.3 of ubiquitin against 0.4 of beta actin and 0.5 of *GAPDH* indicates a stable expression. In general, up-regulation and also down-regulation of genes of interest were noticed after administration of different volumes of DMSO. The level of gene expression was evaluated in all administered volumes. In gene *CYP1A5* statistically significant up-regulation was observed in volumes from 15 to 50 µL. The highest gene overexpression was present in volume of 50 µL (up to 3-fold) and then fold change decreased with the decrease of administered dose of DMSO. For *CYP3A37* statistically significant up-regulation of gene expression can be observed for the volumes from 10 to 50 µL but fold-change was not so high in comparison to *CYP1A5*. For volume 50 µL, approximately 2-fold higher up-regulation of gene expression was noticed. For *CYP3A80*, statistically significant up-regulation was observed in volumes from 20 to 50 µL. The highest fold change was observed in volume 50 µL (up to 1.5-fold). Statistical analysis revealed statistically significant down-regulation for volume 5 µL (Figure 9) in all studied genes of interests (*CYP1A5*, *CYP3A37*, and *CP3A80*).

As a summary, the strongest effect of DMSO on gene expression in the form of up-regulation was observed in gene *CYP1A5*, followed by *CYP3A37* and the lowest up-regulation was present in gene *CYP3A80*. Down-regulation of gene expression for all genes of interest in administered dose 5 µL in comparison to control samples with statistically significant level was observed in all studied genes.

## 4. Discussion

DMSO belongs to xenobiotics with wide spectrum of positive, but also negative effects. Therefore, its use benefits are not clear. DMSO is commonly used as chemical solvent and free radical scavenger. DMSO is also one of the key dipolar aprotic solvents and is less toxic than other members of this class. Numerous in vitro and in vivo experiments focus on the effect of substances dissolved in DMSO, but the toxic effect of DMSO is not monitored in general. The acute toxic effect of DMSO has been studied in mice, rats, rabbits and dogs. 50% DMSO diluted with saline solution was administered to subjects orally, intravenously, intraperitoneally, or subcutaneously. The toxic effect of 50% DMSO was immediate but mild. Typical symptoms that occurred were stiff tail, hypothermia and significant secretion of salivary glands. A diuretic effect was also observed in mice and rats. In rabbits, increased cardiac activity was observed with a marked increase in blood pressure. Weight loss was also observed in the experimental animals. Microscopic examination revealed hepatocyte necrosis and inflammation of the portal system [34].

Our study was focused on the monitoring of DMSO effect on the development of chicken embryo and the liver using various volumes of DMSO (5, 10, 15, 20, 25, 30, 35, 50 µL) administered on ED 4. DMSO has been found to interfere with the cell cycle, affect cell proliferation and differentiation [35]. It may lead to damage to mitochondrial membrane potential, the release of cytochrome c (from mitochondria), and activation of caspases 9 and 3 [36]. One of the possible mechanisms of action of DMSO is thought to be the change in the concentration of cytoplasmic Ca^2+^. Calcium concentration is strictly regulated. The endoplasmic reticulum (ER) is a major site of calcium storage and is essential in maintaining its homeostasis in cells. Organelles such as the ER and mitochondria are functionally closely related. This was also confirmed by studies of the effect of xenobiotics, where morphological and functional changes on these organelles were observed. The release of Ca^2+^ from the ER into the cytoplasm had a direct effect on mitochondrial function [37,38,39,40]. Kang et al. (2017) observed that DMSO significantly increased Ca^2+^ levels in mitochondria and at the same time caused mitochondrial depolarization and dysfunction. This was also reflected in the ultrastructure of mitochondria, which were significantly dilated. In addition to mitochondrial dilatation, fat droplets accumulated in the hepatocytes. According to some literature data, fat droplets are among the most dynamic organelles [40,41]. They play an important role in lipid metabolism and cell homeostasis. They are a reservoir of lipids that provide free fatty acids in the process of lipolysis. These can subsequently use mitochondria as a substrate in β-oxidation, in the formation of ATP, or in ER in the synthesis of phospholipids. The causes of fat droplet accumulation can be various. In our experiment, their increasing amount in hepatocytes may have been caused by impaired mitochondrial function, as described by other authors [42]. The increasing concentration of DMSO on the 3rd day of incubation first caused dilatation of the mitochondria and later damaged the baptisms. As the intensity of mitochondrial damage increased, so did the number of fat droplets in hepatocytes. Because mitochondria are organelles that supply energy for metabolic processes in cells and regulate the amount of Ca^2+^ in the cytoplasm, damage to their structure has a significant effect not only on their function but also on the function of the whole cell. The morphological changes we observe may also be related to the formation of oxygen radicals, which play an important role in embryonic development. Although DMSO is one of the substances with a significant antioxidant effect, work has appeared which also describes its pro-oxidizing properties [43,44]. Kang et al. (2017) observed an imbalance between pro-oxidants and antioxidants due to DMSO. Disruption of this balance has led to an increase in the expression of genes associated with the formation of so-called “unfold” proteins. Endoplasmic-reticular stress (ER stress), mitochondrial dysfunction, and cell apoptosis occurred. Mitochondria are known to be major producers of reactive oxygen species (ROS). Disruption of the balance between ROS production and degradation can lead to oxidative stress. Increasing amounts of radicals can cause damage to DNA, proteins, cytoplasmic membrane, etc. [45]. Recent studies have confirmed that lipid droplets are also essential components of the cellular stress response. Their dynamical synthesis and breakage are interconnected to cellular need and environmental signals. Their biogenesis is induced not only in cell when the cell is exposed to excess amount of lipids, but also by oxidative stress. The sequestration of excess lipids and delayed release of lipids is particularly important for cells exposed to rapidly changing conditions of nutrient and oxidative stress. Enhanced lipid droplet accumulation has been observed in various types of cells including hepatocytes as a mitochondria protection mechanism from lipotoxic damage and for storing lipids for future consumption. They are involved in the regulation of distribution and consumption of lipids during stress in order to maintain energy and redox homeostasis. In cells exposed to oxidative stress, lipid droplets accumulate for protection of membrane from peroxidation reaction, maintain membrane saturation and organelle homeostasis, and enable a long-term supply of lipids for energy production and cell survival [46,47,48].

In general, DMSO is considered as genetically inactive and based on this is frequently used as a solvent in drug-screening assay. However, based on some studies, it is obvious that DMSO in low concentration can alter not only the phenotypic characteristic of hepatic cells but also induce significant alteration of gene expression, protein content and functionality of differentiated hepatic or cardiac cells. DMSO possesses the capability to induce changes in cellular processes within the cell, including alteration in miRNA and epigenetic landscape [2,49]. The results of this alteration can be detected as up or down-regulation of genes. In our study, using molecular analysis of gene expression, we have observed that gene expression increased in direct proportion to the increasing volume of applied DMSO. DMSO induced the gene expression of the *CYP1A5* gene to the highest extent, followed by the *CYP3A37* gene and the least induced expression of the *CYP3A80* gene. A very interesting fact is that, in all genes, it is possible to see significant down regulation using DMSO in volume 5 µL. The increased expression of the *CYP1A5* gene may be related to the fact that DMSO is able to activate the aryl hydrocarbon receptor (AhR), which functions as a ligand-activated transcription factor regulating gene expression. AhR is also referred to as a regulator of gene expression of enzymes metabolizing xenobiotics [50]. Exposure to DMSO activates AhR and induces translocation of AhR to the nucleus and its binding to the target gene promoter, resulting in increased gene expression [51]. Using DMSO may pose a threat, because genome-wide hyper-methylation induced by deregulation of methylation mechanisms may have negative consequences directly, later in life or possibly in a later generation through affecting genes important in development or drug detoxification [2,49].

A large family of ligand-modulated transcription factors involves nuclear receptors mediating cellular responses to small lipophilic molecules, including steroids, retinoids, fatty acids, and exogenous ligands. For the regulation of drug-mediated induction of cytochrome P450 (*CYP*) as a major drug metabolizing enzyme, orphan nuclear receptors with no known endogenous ligands have been discovered. *CYP3A37* and *CYP3A80* isoforms belong to the group of *CYP3A* cytochromes occurring mainly in the liver and intestine. A wide range of compounds such as antibiotics, glucocorticoids or pesticides can induce their expression. It has been proven that DMSO increases the expression of nuclear receptors PXR and CAR, which is associated with increased expression of cytochromes, mainly from *CYP3A* [52]. The nuclear receptors CAR and PXR belong as AhR to transcription factors that are activated by ligands (e.g., xenobiotics such as DMSO) [53]. When a ligand binds to a nuclear receptor, the nuclear receptor is activated, following by binding to the DNA promoter to start the gene transcription. In our study, there was an increase in gene expression in the liver tissue of two cytochrome P450 groups (*CYP1A* and *CYP3A*) after DMSO administration. Chicken xenobiotic receptor (CXR) closely related to human pregnant X receptor (PXR) and constitutive androstane receptor (CAR) may be involved in the activation of expression of *CYP3A* isoforms. The expression of CXR is restricted to tissues where drug induction of CYPs predominantly occurs (liver, kidney, small intestine, and colon). Phenobarbital-responsive enhancer unit (PBRU) in the 5’-flanking region of the chicken *CYP2H1* gene is necessary for CXR binding. In CV-1 monkey cell transactivation assays, a variety of chemicals, drugs and steroids are responsible for activation of CXR. In a chicken hepatoma cell line, the same agents trigger the induction of PBRU-dependent reporter gene expression and *CYP2H1* transcription. Based on these outputs, it is evident, that CXR belongs to the family of xenobiotic-activated orphan nuclear receptor with a major role in the regulation of *CYP2H1*. These results provide convincing evidence for a major role of CXR in the regulation of *CYP2H1* and add a member to the family of xenobiotic-activated orphan nuclear receptors [54]. Cytochrome activity and expression is a major determinant of drug efficacy and toxicity. When monitoring the activity of detoxifying enzymes in different species of poultry, large differences in the kinetics of enzymes were found [6]. The different susceptibility of individual species to some xenobiotics can be partly explained by differences in the relative enzymatic properties of cytochromes.

## 5. Conclusions

The chicken embryo as an alternative animal model is used within 3Rs principles for the testing of hydrophobic substances, which require DMSO as a solvent or penetrating vehicle for different drugs. Regarding our results, the applicable volume of DMSO is limited, and it can affect chicken embryonic development at doses higher than 10 µL (significant reduction of body weight, the occurrence of malformations). Current findings suggested that exposure to DMSO caused histopathological alterations in the developing liver using the volume of DMSO higher than 25 µL. Based on the results of cytochrome P450 complex (*CYP1A5*, *CYP3A37*, *CYP3A80*) gene expression, it is hard to talk about the safe dose of DMSO. Not only could the up-regulation of selected genes was observed, but significant down-regulation was also present with the lowest administered volume 5 µL of DMSO. Even the histopathological or morphological changes are not detectable after administration of the lowest volume of DMSO, the gene expression analysis in our study showed that DMSO could affect hepatocytes at the molecular level. These findings should be taken into account when the avian developing model is used for the testing of various substances dissolved in DMSO, mainly during its early stage of development. Assuming that the use of DMSO is unavoidable in biological research, it is necessary to keep the working concentration as low as possible to not affect internal homeostasis of cells.

## Figures and Tables

**Table 1:** ED 9 embryotoxicity due to DMSO injection on ED 4.

Dose (µL)	N	Dead	Mortality (%)	Malformations	Mean Body Weight (g)	Body Weight SD
0	12	0	0	0	2.185	0.316
5	11	0	0	0	2.126	0.241
10	12	3	25	0	**1.886**	0.142
15	12	4	33	1	**1.779**	0.091
20	12	5	42	0	**1.285**	0.150
25	12	5	42	5	**0.918**	0.252
30	12	5	42	6	**0.911**	0.164
35	12	9	75	3	**0.880**	0.069
	**95**	**31**	**33**	**15**		

Wet weight of embryos sampled on ED 9. Differences in body weight considered statistically significant are in bold (*p* < 0.01) N–total number of embryos per group; SD—standard deviation. The values of mortality are used for construction of Figure 1. The weight of embryos at ED 4 according to Clark et al. (1986) is 80 mg [32].

## Data Availability

The data presented in this study are available on request from the corresponding author. The data are not publicly available due to the fact that these data are published for the first time and authors have no problems to provide them on request.

## References

[1] Galvao J., Davis B., Tilley M., Normando E., Duchen M.R., Cordeiro M.F. (2014). Unexpected low-dose toxicity of the universal solvent DMSO. FASEB J..

[2] Verheijen M., Lienhard M., Schrooders Y., Clayton O., Nudischer R., Boerno S., Timmermann B., Selevsek N., Schlapbach R., Gmuender H. (2019). DMSO induces drastic changes in human cellular processes and epigenetic landscape in vitro. Sci. Rep..

[3] Chen T.-H., Wang Y.-H., Wu Y.-H. (2011). Developmental exposures to ethanol or dimethylsulfoxide at low concentrations alter locomotor activity in larval zebrafish: Implications for behavioral toxicity bioassays. Aquat. Toxicol..

[4] Madsen B.K., Hilscher M., Zetner D., Rosenberg J. (2018). Adverse reactions of dimethyl sulfoxide in humans: A systematic review. F1000 Res..

[5] Watanabe K.P., Kawai Y.K., Ikenaka Y., Kawata M., Ikushiro S.-I., Sakaki T., Ishizuka M. (2013). Avian Cytochrome P450 (CYP) 1-3 Family Genes: Isoforms, Evolutionary Relationships, and mRNA Expression in Chicken Liver. PLoS ONE.

[6] Zanger U.M., Schwab M. (2013). Cytochrome P450 enzymes in drug metabolism: Regulation of gene expression, enzyme activities, and impact of genetic variation. Pharmacol. Ther..

[7] Gannon M., Gilday D., Rifkind A.B. (2000). TCDD Induces CYP1A4 and CYP1A5 in Chick Liver and Kidney and Only CYP1A4, an Enzyme Lacking Arachidonic Acid Epoxygenase Activity, in Myocardium and Vascular Endothelium. Toxicol. Appl. Pharmacol..

[8] Lee J.-S., Kim E.-Y., Iwata H. (2009). Dioxin activation of CYP1A5 promoter/enhancer regions from two avian species, common cormorant (*Phalacrocorax carbo*) and chicken (*Gallus gallus*): Association with aryl hydrocarbon receptor 1 and 2 isoforms. Toxicol. Appl. Pharmacol..

[9] Head J.A., Kennedy S.W. (2007). Differential expression, induction, and stability of CYP1A4 and CYP1A5 mRNA in chicken and herring gull embryo hepatocytes. Comp. Biochem. Physiol. Part C Toxicol. Pharmacol..

[10] Watanabe M.X., Jones S.P., Iwata H., Kim E.-Y., Kennedy S.W. (2009). Effects of co-exposure to 2,3,7,8-tetrachlorodibenzo-p-dioxin and perfluorooctane sulfonate or perfluorooctanoic acid on expression of cytochrome P450 isoforms in chicken (*Gallus gallus*) embryo hepatocyte cultures. Comp. Biochem. Physiol. Part C Toxicol. Pharmacol..

[11] Fathi M.A., Han G., Kang R., Shen D., Shen J., Li C. (2020). Disruption of cytochrome P450 enzymes in the liver and small intestine in chicken embryos in ovo exposed to glyphosate. Environ. Sci. Pollut. Res..

[12] Yang J., An J., Li M., Hou X., Qiu X. (2013). Characterization of chicken cytochrome P450 1A4 and 1A5: Inter-paralog comparisons of substrate preference and inhibitor selectivity. Comp. Biochem. Physiol. Part C Toxicol. Pharmacol..

[13] Kapelyukh Y., Henderson C.J., Scheer N., Rode A., Wolf C.R. (2019). Defining the Contribution of CYP1A1 and CYP1A2 to Drug Metabolism Using Humanized CYP1A1/1A2 and Cyp1a1/Cyp1a2 Knockout Mice. Drug Metab. Dispos..

[14] Shang S., Jiang J., Deng Y. (2013). Chicken Cytochrome P450 1A5 Is the Key Enzyme for Metabolizing T-2 Toxin to 3’OH-T-2. Int. J. Mol. Sci..

[15] Ourlin J.-C., Baader M., Fraser D., Halpert J.R., Meyer U.A. (2000). Cloning and Functional Expression of a First Inducible Avian Cytochrome P450 of the CYP3A Subfamily (CYP3A37). Arch. Biochem. Biophys..

[16] Yuan Y., Zhou X., Yang J., Li M., Qiu X. (2013). T-2 toxin is hydroxylated by chicken CYP3A37. Food Chem. Toxicol..

[17] Greenblatt D.J., Zhao Y., Venkatakrishnan K., Duan S.X., Harmatz J.S., Parent S.J., Court M.H., von Moltke L.L. (2011). Mechanism of cytochrome P450-3A inhibition by ketoconazole. J. Pharm. Pharmacol..

[18] Fagerberg L., Hallström B.M., Oksvold P., Kampf C., Djureinovic D., Odeberg J., Habuka M., Tahmasebpoor S., Danielsson A., Edlund K. (2014). Analysis of the Human Tissue-specific Expression by Genome-wide Integration of Transcriptomics and Antibody-based Proteomics. Mol. Cell. Proteom..

[19] Uhlén M., Fagerberg L., Hallström B.M., Lindskog C., Oksvold P., Mardinoglu A., Sivertsson Å., Kampf C., Sjöstedt E., Asplund A. (2015). Tissue-based map of the human proteome. Science.

[20] Kawai Y.K., Itou K., Yoshino T., Iima H., Matsumoto F., Kubota A. (2020). Hepatic transcriptional profile and tissue distribution of cytochrome P450 1-3 genes in the red-crowned crane Grus japonensis. Comp. Biochem. Physiol. Part C Toxicol. Pharmacol..

[21] Geng W., Long S.L., Chang Y.-J., Saxton A.M., Joyce S.A., Lin J. (2020). Evaluation of bile salt hydrolase inhibitor efficacy for modulating host bile profile and physiology using a chicken model system. Sci. Rep..

[22] Luo G., Guenthner T., Gan L.-S., Humphreys W.G. (2004). CYP3A4 Induction by Xenobiotics: Biochemistry, Experimental Methods and Impact on Drug Discovery and Development. Curr. Drug Metab..

[23] Doke S.K., Dhawale S.C. (2015). Alternatives to animal testing: A review. Saudi Pharm. J..

[24] Ribatti D., Annese T., Tamma R. (2020). The use of the chick embryo CAM assay in the study of angiogenic activiy of biomaterials. Microvasc. Res..

[25] Kue C.S., Tan K.Y., Lam M.L., Lee H.B. (2015). Chick embryo chorioallantoic membrane (CAM): An alternative predictive model in acute toxicological studies for anti-cancer drugs. Exp. Anim..

[26] Tavakkoli H., Attaran R., Khosravi A., Salari Z., Salarkia E., Dabiri S., Mosallanejad S.S. (2019). Vascular alteration in relation to fosfomycine: In silico and in vivo investigations using a chick embryo model. Biomed. Pharmacother..

[27] Rashidi H., Sottile V. (2009). The chick embryo: Hatching a model for contemporary biomedical research. BioEssays.

[28] Psychoyos D., Finnell R. (2008). Method for Culture of Early Chick Embryos ex vivo (New Culture). J. Vis. Exp..

[29] Kiecker C. (2016). The chick embryo as a model for the effects of prenatal exposure to alcohol on craniofacial development. Dev. Biol..

[30] Merckx M.G., Tay M.H., Monaco M.M.L., van Zandvoort M.A., De Spiegelaere W., Lambrichts I., Bronckaers A. (2020). Chorioallantoic Membrane Assay as Model for Angiogenesis in Tissue Engineering: Focus on Stem Cells. Tissue Eng. Part B Rev..

[31] Sedmera D., Hu N., Weiss K.M., Keller B.B., Denslow S., Thompson R.P. (2002). Cellular changes in experimental left heart hypoplasia. Anat. Rec. Adv. Integr. Anat. Evol. Biol..

[32] Clark E.B., Hu N., Dummett J.L., VandeKieft G.K., Olson C., Tomanek R. (1986). Ventricular function and morphology in chick embryo from stages 18 to 29. Am. J. Physiol. Circ. Physiol..

[33] Zhang L.L., Zhang J.R., Yu Z.G., Zhao J., Mo F., Jiang S.X. (2010). Effects of ionophores on liver CYP1A and 3A in male broilers. J. Vet. Pharmacol. Ther..

[34] Caujolle F.M.E., Caujolle D.H., Cros S.B., Calvet M.-M.J. (1967). Limits of toxic and teratogenic tolerance of dimethyl sulfoxide. Ann. N. Y. Acad. Sci..

[35] Srinivas S., Sironmani T., Shanmugam G. (1991). Dimethyl sulfoxide inhibits the expression of early growth-response genes and arrests fibroblasts at quiescence. Exp. Cell Res..

[36] Liu J., Yoshikawa H., Nakajima Y., Tasaka K. (2001). Involvement of mitochondrial permeability transition and caspase-9 activation in dimethyl sulfoxide-induced apoptosis of EL-4 lymphoma cells. Int. Immunopharmacol..

[37] Pal R., Mamidi M.K., Das A.K., Bhonde R. (2012). Diverse effects of dimethyl sulfoxide (DMSO) on the differentiation potential of human embryonic stem cells. Arch. Toxicol..

[38] Biagioli M., Pifferi S., Ragghianti M., Bucci S., Rizzuto R., Pinton P. (2008). Endoplasmic reticulum stress and alteration in calcium homeostasis are involved in cadmium-induced apoptosis. Cell Calcium.

[39] La Rovere R.M., Roest G., Bultynck G., Parys J.B. (2016). Intracellular Ca^2+^ signaling and Ca^2+^ microdomains in the control of cell survival, apoptosis and autophagy. Cell Calcium.

[40] Kang M.-H., Das J., Gurunathan S., Park H.-W., Song H., Park C., Kim J.-H. (2017). The cytotoxic effects of dimethyl sulfoxide in mouse preimplantation embryos: A mechanistic study. Theranostics.

[41] Welte M.A. (2007). Proteins under new management: Lipid droplets deliver. Trends Cell Biol..

[42] Murphy S., Martin S., Parton R.G. (2009). Lipid droplet-organelle interactions; sharing the fats. Biochim. Biophys. Acta Mol. Cell Biol. Lipids.

[43] Goto Y., Noda Y., Mori T., Nakano M. (1993). Increased generation of reactive oxygen species in embryos cultured in vitro. Free Radic. Biol. Med..

[44] Sadowska-Bartosz I., Pączka A., Mołoń M., Bartosz G. (2013). Dimethyl sulfoxide induces oxidative stress in the yeast Saccharomyces cerevisiae. FEMS Yeast Res..

[45] He L., He T., Farrar S., Ji L., Liu T., Ma X. (2017). Antioxidants Maintain Cellular Redox Homeostasis by Elimination of Reactive Oxygen Species. Cell. Physiol. Biochem..

[46] Jarc E., Petan T. (2019). Lipid Droplets and the Management of Cellular Stress. Yale J. Biol. Med..

[47] Rambold A.S., Cohen S., Lippincott-Schwartz J. (2015). Fatty Acid Trafficking in Starved Cells: Regulation by Lipid Droplet Lipolysis, Autophagy, and Mitochondrial Fusion Dynamics. Dev. Cell.

[48] Nguyen T.B., Louie S.M., Daniele J.R., Tran Q., Dillin A., Zoncu R., Nomura D.K., Olzmann J.A. (2017). DGAT1-Dependent Lipid Droplet Biogenesis Protects Mitochondrial Function during Starvation-Induced Autophagy. Dev. Cell.

[49] Tunçer S., Gurbanov R., Sheraj I., Solel E., Esenturk O., Banerjee S. (2018). Low dose dimethyl sulfoxide driven gross molecular changes have the potential to interfere with various cellular processes. Sci. Rep..

[50] Fujii-Kuriyama Y., Mimura J. (2005). Molecular mechanisms of AhR functions in the regulation of cytochrome P450 genes. Biochem. Biophys. Res. Commun..

[51] Zhang H.F., Lin X.H., Yang H., Zhou L.C., Guo Y.L., Barnett J.V., Guo Z.M. (2012). Regulation of the Activity and Expression of Aryl Hydrocarbon Receptor by Ethanol in Mouse Hepatic Stellate Cells. Alcohol. Clin. Exp. Res..

[52] Su T., Waxman D.J. (2004). Impact of dimethyl sulfoxide on expression of nuclear receptors and drug-inducible cytochromes P450 in primary rat hepatocytes. Arch. Biochem. Biophys..

[53] Honkakoski P., Negishi M. (2000). Regulation of cytochrome P450 (CYP) genes by nuclear receptors. Biochem. J..

[54] Handschin C., Podvinec M., Meyer U.A. (2000). CXR, a chicken xenobiotic-sensing orphan nuclear receptor, is related to both mammalian pregnane X receptor (PXR) and constitutive androstane receptor (CAR). Proc. Natl. Acad. Sci. USA.

